# Transesophageal pacing : a versatile diagnostic and therapeutic tool

**Published:** 2003-10-01

**Authors:** Thierry Verbeet, José Castro, Pierre Decoodt

**Affiliations:** Department of Cardiology, University Hospital Brugmann, Brussels, Belgium

**Keywords:** transesophageal pacing, esophageal pacing, atrial pacing, supraventricular arrhythmias

## Introduction

Transesophageal atrial pacing is feasible because of the proximity between the oesophagus and the posterior aspect of the atria [1]. Atrial pacing is possible through the oesophagus in almost all patients and can yield important information in many arrhythmias where single site atrial pacing is of value. It is a cheap procedure. There is no need for X Rays or cathlab.

## Technical considerations

Transesophageal pacing and recording is done using specialized or non specialized catheters. There are two different lead types: 1) the pill electrode, connected to a flexible wire, that the patient swallows with water. This pill electrode necessitates patient collaboration. 2) a flexible catheter that can even be used in comatose or intubated patients.

The pacing lead, in our cases a bipolar flexible catheter, is introduced into the oesophagus via the nares after local nose anesthesia with xylocaine spray. Sometimes throat anesthesia with the same spray is also performed. Xylocaine gel is used to lubricate the lead and the lead is introduced with guidewires in it in order to increase its stiffness. It is positioned into the oesophagus in order to record the posterior paraseptal atrial electrogram. There is a relationship between the site of maximal atrial amplitude and the lowest atrial pacing threshold. The optimal atrial pacing site is usually found around 40 cm from the nares. Bipolar or unipolar recordings can be made.

The strengh-duration curve of esophageal pacing is different than that of endocardial pacing. The lowest thresholds can be reached at pulse widths between 10 and 20 msec. Thresholds at these pulse widths are usually situated between 5 and 15 mA [2]. So transesophageal pacing necessitates a specific pacing device and external pacemakers cannot be used for that purpose.

Commercially available transesophageal pacing systems are usually restricted to burst pacing only. They frequently have an input where one can connect a programmable stimulator that will trigger esophageal pacing enabling the delivery of extrastimuli.

**Figure 1:**
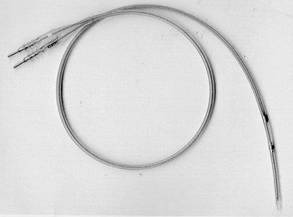
Medtronic 6992A lead

**Figure 2:**
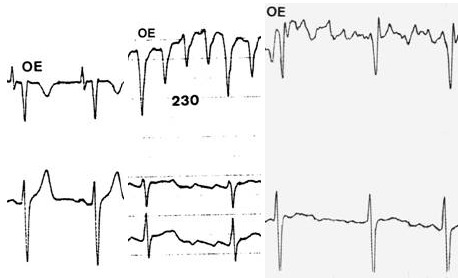
Esophageal electrograms in sinus rhythm (left), atrial flutter (center) and atrial fibrillation (right)

The tracings shown were all obtained in our center using the following equipment: a Medtronic 6992A bipolar pacing lead (Figure 1) initially designed to pace the coronary sinus on a permanent basis, a dedicated A.P.I. Electronique Oesostim 2 transesophageal stimulator able to provide a maximal pulse width of 22 ms and a maximal current intensity of 44 mA, an implantable programmable pacemaker Medtronic Kappa KDR 700 that is connected to the ‘synchronisation input’ of the esophageal stimulator in order to deliver a maximum of 4 programmable extrabeats using the non invasive programmable stimulation features of this pacemaker model. The pacemaker is used as a triggering system because the dedicated esophagal stimulators can usually deliver bursts only. In fact any pacemaker with non invasive programmed stimulation possibilities will do. Alternatively a classic programmable stimulator can also be used instead. Intraesophageal electrograms are usually simply recorded in unipolar mode via a classic precordial EKG lead but can also be recorded in bipolar mode using limb leads (Figure 2) [3].

Due to the bipolar nature of our lead, pacing and recording cannot be obtained simultaneously. If this is mandatory quadripolar catheters have to be used. The patient is asked to fasten for 4 hours before the procedure and for as long as the throat anesthesia remains.

## Applications

Transesophageal pacing can yield important information in many situation where invasive atrial stimulation is frequently done [4] [11].

**Figure 3:**
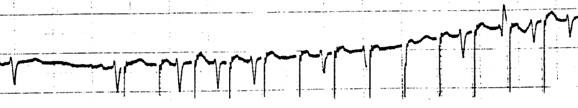
Evaluation of AV nodal Wenckebach point with esophageal pacing under betablocking therapy in a patient suffering of fast conducting atrial fibrillation

**Figure 4:**
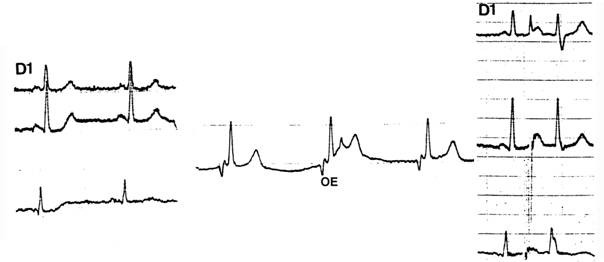
This patient complains of palpitations and has a short PR that could mask a delta wave. A single esophageal extrastimulus (right) captures the atrium, prolongs the atrioventricular conduction and does not demonstrate any preexcitation.

**Figure 5:**
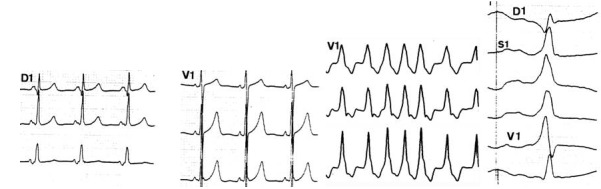
This patient presents with atrial fibrillation conducting along a left lateral accessory pathway (third set), although preexcitation is concealed on the sinus surface EKG due to rapid atrioventricular conduction and short PR (first two sets). Atrial pacing during the ablation session unmasks preexcitation (fourth set). This emphasizes the usefulness of atrial pacing in short PR accompanied by palpitations.

**Figure 6:**
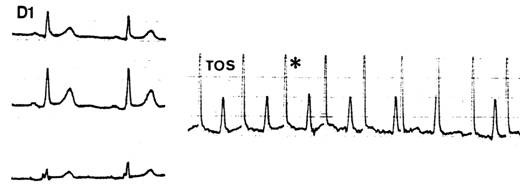
Assessment of initial slurring of the QRS complex. Preexcitation is excluded by esophageal pacing that demonstrates prolongation of atrioventricular nodal conduction without preexcitation.

**Figure 7:**
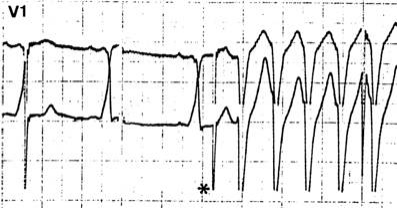
Esophageal pacing triggers a reciprocating tachycardia with LBBB aberration in a patient with WPW disease.

**Figure 8:**
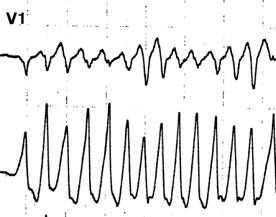
Same patient: atrial fibrillation with fast conducting ventricular conduction occurs through the accessory pathways after an attempt to stop the reciprocating tachycardia with esophageal pacing. Induction of atrial fibrillation is also useful when an esophageal stimulus is not followed by a QRS complex and atrial capture is difficult to assess on the surface EKG.

**Figure 9:**
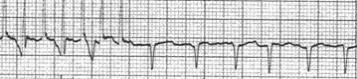
Induction of atrial fibrillation with esophageal pacing in a patient with WPW disease. QRS complexes during atrial fibrillation are not preexcited demonstrating poor anterograde accessory pathway conduction.

**Figure 10:**
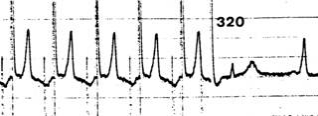
Assessment of anterograde accessory pathway refractory period (in this case 320 msec) in a patient with WPW disease using transesophageal pacing.

**Figure 11:**
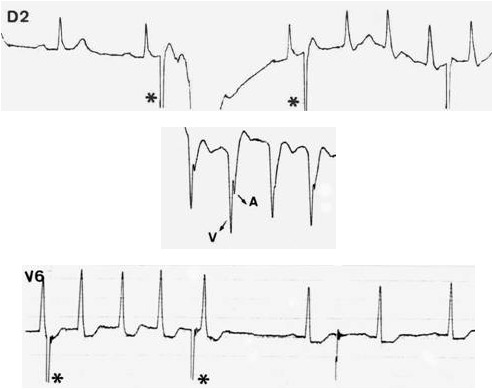
Triggering of AV nodal reentrant tachycardia (above) in a patient with undocumented episodes of palpitations. The esophageal electrogram (middle) shows fused atria and ventricular electrograms. The surface EKG during tachycardia is also diagnostic (see above, pseudo S waves in D2). The tachycardia is stopped by one esophageal stimulus (below).

**Figure 12:**
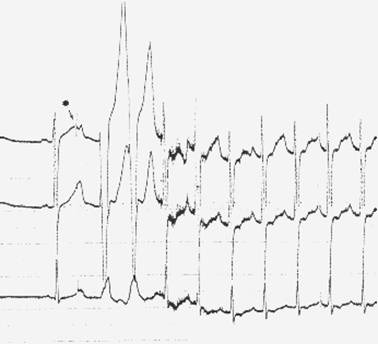
Triggering of reciprocating tachycardia with a single esophageal stimulus in a patient with undocumented palpitations

**Figure 13:**
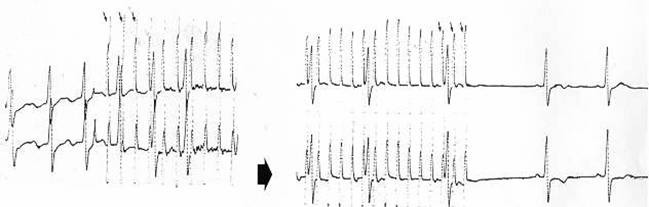
Interruption of typical atrial flutter with esophageal pacing

- Sinus node evaluation

- Atrioventricular conduction evaluation: permeability, short PR, effects of drugs (Figure 3-6)

- Assessment of Woff-Parkinson-White syndrome: reciprocating tachycardia inducibility, anterograde refractory period, effect of drugs, ventricular rate during atrial fibrillation (Figure 7-10)

- Assessment of paroxysmal supraventricular tachycardia of unknown origin: mechanism analysis, counselling for radiofrequency ablation, drug evaluation.

- Assessment of palpitations of unknown origin (Figure 11-12)

- Assessment of relationship between atrium and ventricle: differential diagnosis ventricular tachycardia versus supraventricular tachycardia.

- Interruption of supraventricular tachycardia and atrial flutter (Figure 13)

- Miscellaneous: P wave synchronous pacing, evaluation of myocardial ischemia

There are a few limitations to the technique: a) there is only one site of atrial pacing and recording, b) there is no ventricular pacing, c) sometimes atrial capture can be difficult to assess on the surface ekg during premature extrastimuli delivery.

**Figure 14:**
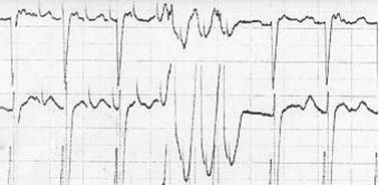
Inadvertent ventricular pacing occurs when the lead is pushed too deep in the oesophagus and high energies are used (PW: 20 ms, intensity 15 mA)

Transesophageal atrial pacing can be obtained in more than 95 % of patients. Esophageal pacing usually produces a burning chest sensation that most patients tolerate. Bust pacing is always started at a low rate to educate the patient to this burning sensation and also to avoid rapid ventricular pacing which may very rarely occur (Figure 14). Brachial plexus stimulation and phrenic nerve pacing although reported in the literature have never been seen in our center.

Between 1991 and 1997, 51 patients were treated with esophageal pacing for atrial flutter mostly of the common form. In 37 % of patients sinus rhythm was immediately established. In 16 % patients sinus rhythm was established after conversion of atrial flutter to atrial fibrillation. The technique failed in 47 % patients either because the patients remained in iatrogenic atrial fibrillation or because they went back from atrial fibrillation to flutter. This has led us to propose external defibrillation as a first line therapy for acute conversion in these patients except in selected cases [12].

## Conclusions

Transesophageal pacing is a versatile tool and may replace invasive atrial pacing in many circumstances. In the vast majority of cases where a preablation measurement of electrophysiological properties is needed it could avoid performing separate diagnostic and curative procedures.

## References

[R1] Prystowsky EN, Pritchett ELC, Gallagher JJ (1980). Origin of the atrial electrogram recorded from the esophagus. Circulation.

[R2]  Benson DW, Sanford M, Dunnigan A (1984). Transesophageal atrial pacing threshold: role of interelectrode spacing, pulse width, and catheter insertion depth. Am J Cardiol.

[R3] Hammill SC, Pritchett ELC (1981). Simplified esophageal electrocardiography using bipolar recording, leads. Ann Intern Med.

[R4] Gallagher JJ, Smith WM (1982). Esophageal pacing: a diagnostic and therapeutic tool. Circulation.

[R5] Critelli G, Grassi G, Perticone F (1983). Transesophageal pacing for prognostic evaluation of preexcitation syndrome and assessment of protective therapy. Am J Cardiol.

[R6] Gallagher JJ, Smith WM, Kasell J (1980). The use of the esophageal lead in the diagnosis of mechanisms of reciprocating supraventricular tachycardia. PACE.

[R7] Falk RH, Werner M (1987). Transesophageal atrial pacing using a pill electrode for the termination of atrial flutter. Chest.

[R8] Benson DW, Dunnigan A, Sterba R (1983). Atrial pacing from the esophagus in the diagnosis and management of tachycardia and palpitations. J Pediatr.

[R9] Burack B, Furman S (1969). Transesophageal cardiac pacing. Am J Cardiol.

[R10] Lubell DL (1971). Cardiac pacing from the esophagus. Am J Cardiol.

[R11]  Brunetto JF, Sgammini HO, Ledesma RE (1979). Evaluation of sinoatrial node function through the use of transesophageal atrial pacing (Abstract). PACE.

[R12] Verbeet T, Castro J, Morissens M (1978). Transesophageal pacing, a useful tool in the management of arrhythmias (abstract). Archives des Maladies du Coeur et des Vaisseaux.

